# Pathways to labor engagement: young people’s experiences of social prescribing as a response to the NEET phenomenon

**DOI:** 10.3389/fpubh.2026.1778162

**Published:** 2026-05-25

**Authors:** Isabel Farina, Cristina Masella, Marcello Bertotti

**Affiliations:** 1 Department of Management Engineering, Politecnico of Milan, Milan, Italy; 2 Institute for Connected Communities, University of East London, London, United Kingdom

**Keywords:** labor engagement, mental wellbeing, NEET youth, psychosocial readiness, social prescribing

## Abstract

**Background:**

Young people Not in Education, Employment or Training (NEET) constitute a heterogeneous population facing intertwined psychological, social and structural barriers to labor market participation. Disengagement from work is often linked to mental health difficulties, social isolation and reduced agency. Although social prescribing has gained attention as a public health approach to address social determinants of health, its role in supporting employment pathways remains underexplored, particularly for young people in NEET situations.

**Methods:**

This qualitative study explores young people’s experiences of the C.O.P.E. social prescribing intervention implemented in the Autonomous Province of Trento, Italy. Semi-structured interviews were conducted with 15 participants aged 16–34 (in line with the extended NEET definition used in Italian policy frameworks), selected through maximum variation sampling to capture diversity in age, gender, educational background and levels of social isolation. Data were analyzed using inductive thematic analysis.

**Results:**

Findings highlight the heterogeneity of NEET trajectories and a strained relationship with the labor market, characterized by fear, low self-confidence and disorientation. Participants described the intervention not as a direct route to employment, but as a relational and preparatory space supporting trust-building, emotional stabilization and gradual re-engagement. The Link Worker emerged as a key ‘in-between’ figure, combining practical guidance with person-centered support. Reported outcomes included increased self-esteem, agency and clarity about future steps, rather than immediate employment.

**Conclusion:**

From users’ perspectives, social prescribing may support psychosocial readiness for labor market engagement rather than direct employment outcomes. The findings suggest that policies addressing youth disengagement should integrate relational and wellbeing-oriented support with activation and employment projects and services. Embedding social prescribing within coordinated health and labor systems may improve engagement among vulnerable young people.

## 1 Introduction

### 1.1 The NEET issue

The term NEET (Not in Education, Employment or Training) is commonly used to describe young people who are not engaged in formal education, training, or paid employment. First introduced in the United Kingdom in the late 1990s (1) to address youth disengagement from school and the labor market, the concept was later adopted at European level as a policy indicator to guide youth employment strategies. Over time, the age range associated with the NEET category has expanded, reflecting both demographic changes and the growing recognition that prolonged inactivity increasingly affects young adults well beyond adolescence (2). Recent research on school-to-work transitions further highlights how fragmented and delayed trajectories are becoming more common in contexts characterized by high rates of youth disengagement, reflecting structural constraints and labor market instability (3).

Despite its widespread use, the NEET category does not describe a homogeneous group. Rather, it encompasses a highly diverse population, characterized by different trajectories, resources and levels of vulnerability. Definitions vary across national contexts, but in general NEET status refers to young people who, regardless of their educational attainment, experience a period of disconnection from both education and employment, often accompanied by an increased risk of social and labor market exclusion (4). This condition may be influenced by a combination of individual factors—such as migration background, disability, low educational attainment or socioeconomic disadvantage—and broader structural factors, including labor market conditions and welfare systems (5). Emerging evidence also emphasizes how socioeconomic inequalities and institutional arrangements shape NEET trajectories, reinforcing cumulative disadvantage over time (5). More recent scholarship has critically examined the NEET construct, arguing that it risks homogenizing diverse trajectories and reproducing stigmatizing interpretations of youth inactivity, while overlooking relational and institutional dynamics (6). This shift reflects an effort to challenge simplified and often stigmatizing representations of NEETs as disengaged by choice or lacking motivation (7). Instead, recent studies emphasize the relational and structural dimensions of inactivity, highlighting how limited access to social networks, weak institutional trust and experiences of marginalization shape young people’s trajectories (8).

Mental health plays a crucial role within this framework. Evidence consistently shows that being in a NEET condition is associated with higher risks of depression, anxiety, substance use and suicidal ideation (9). Vocational disengagement and mental health difficulties are often interconnected, reinforcing each other over time and contributing to prolonged exclusion from education and employment (10). A recent study found that youths who remain in the ‘NEET trap’ are doubly disadvantaged not only in suffering socioeconomic exclusion but also in missing out on the potential benefits of mental health early intervention (11, 12).

As a result, NEET status should be understood not only as an employment-related issue, but also as a broader condition affecting young people’s wellbeing, agency and capacity to re-engage with social institutions (13).

From a policy perspective, the challenge lies in designing interventions capable of reaching and engaging those young people who experience the most persistent forms of disengagement. Recent research has framed the NEET phenomenon as a broader socioeconomic issue, highlighting its systemic implications for labor markets and social cohesion (14). At European level, the Youth Guarantee represents the main policy response aimed at facilitating young people’s transition into employment or training. While the program has expanded access to employment-related support, recent evidence suggests that structural labor market conditions and institutional fragmentation continue to limit its effectiveness, particularly for the most marginalized (15). In Italy, despite significant investment, structural limitations of employment services and fragmented governance have further constrained the program’s capacity to address the diversity of NEET experiences (16, 17).

This study is informed by a conceptual perspective that understands NEET status as the outcome of an interaction between structural, relational and individual dimensions. Structural factors include labor market conditions and institutional arrangements, which shape opportunities and constraints for youth transitions. Relational dimensions refer to access to social networks, trust in institutions and the quality of support systems. Individual factors include mental health, self-efficacy and agency. These dimensions are deeply interconnected: structural barriers may weaken relational resources, while relational disconnection can undermine individual capacity to engage with education and employment. Within this framework, social prescribing can be conceptualized as an intervention that operates primarily at the relational level, with potential indirect effects on individual agency and, over time, on labor market engagement.

### 1.2 Social prescribing to address young people’s needs and employment

Social Prescribing is defined as “‘a means for trusted individuals in clinical and community settings to identify that a person has non-medical, health-related social needs and to subsequently connect them to non-clinical supports and services within the community by co-producing a social prescription—a non-medical prescription, to improve health and well-being and to strengthen community connections” (18). It has increasingly been promoted as an approach capable of addressing the social determinants of health by connecting individuals with community-based resources (19). Within this framework, employment is widely recognized as a key determinant of both physical and mental health, contributing to financial security, social identity and psychological wellbeing. At the same time, however, social prescribing interventions have largely developed within the health sector, where work-related goals are often treated as secondary or even external to the primary mission of improving health (20). This tension constitutes a central paradox: while employment is acknowledged as fundamental to wellbeing, it remains marginal within the practical implementation of social prescribing (21).

Existing social prescribing models have predominantly focused on addressing social isolation, loneliness and mental health difficulties, particularly among adult populations (22–24). Referrals typically originate from primary care, reinforcing a clinical framing of need and intervention. Although policy discussions—particularly in the United Kingdom—have increasingly highlighted the potential of employment as a pathway to improved health, empirical evidence suggests that work-related outcomes are rarely prioritized in practice (25, 26). At the same time, social prescribing has expanded across several European countries, including the Netherlands, Spain, Austria and the Nordic region, where models vary in terms of institutional embedding, target populations and integration with social and employment services (27–29). In many of these contexts, social prescribing is implemented through diverse governance arrangements, reflecting differences in welfare systems and policy priorities. Overall, services tend to prioritize the stabilization of psychosocial needs, with employment framed as a longer-term and indirect outcome.

This dynamic is especially relevant when considering young people in NEET conditions. For this population, disengagement from education and employment is often intertwined with mental health difficulties, low self-confidence and limited social networks. While work can represent an important source of recovery, structure and social inclusion, young NEETs frequently lack the readiness or resources to engage with conventional employment services. As a result, interventions that focus exclusively on rapid labor market activation risk reproducing cycles of exclusion and disengagement.

Social prescribing occupies an ambiguous position in this context. On the one hand, its emphasis on personalized support, trust-building and relational proximity offers a potentially effective means of engaging young people who are distant from institutional services. On the other hand, comparative evidence suggests that social prescribing models differ significantly across welfare systems, ranging from highly institutionalized, health-led approaches (such as in the UK) to more community-based and socially embedded models in other European contexts, where links with employment and social services may be more explicitly developed (30–32). However, the predominant health-oriented framing of social prescribing continues to limit its capacity to explicitly address employment-related needs. Link workers often lack specific training or mandates to support pathways to work, and existing models vary widely in their ability to integrate employment-related resources in a coherent manner.

Social prescribing continues to be primarily situated within the health domain, reinforcing a conceptual and operational separation between wellbeing-oriented support and labor market interventions. While this positioning has contributed to expanding non-clinical and community-based responses to health-related needs, it also risks overlooking the interdependence between health and employment, particularly for young people whose inactivity is shaped by a combination of psychosocial vulnerabilities and structural barriers. For young people in NEET situations, difficulties related to mental health, low self-confidence and social isolation often coexist with fragmented educational trajectories and limited access to meaningful employment opportunities.

Emerging evidence from recent evaluations of social prescribing initiatives targeting young people in NEET conditions (33) suggests that the value of these interventions may lie less in direct or immediate employment outcomes and more in their capacity to function as enabling spaces. Rather than acting as linear pathways into work, social prescribing interventions appear to support relational engagement, the development of agency, confidence and social capital, and a gradual re-connection with institutional and community contexts. These processes may represent important preconditions for later engagement with education or employment, challenging narrow or instrumental interpretations of labor market activation.

Against this backdrop, examining social prescribing initiatives that explicitly target young people in NEET situations offers an opportunity to critically interrogate the health–work paradox. The aim of this study is to explore how young people in NEET situations experience a social prescribing intervention and how such interventions may support psychosocial readiness for labor market engagement. By focusing on users’ perspectives, the study examines how relational forms of support may contribute to processes of gradual re-engagement with social and employment pathways, without reducing employment to a singular or short-term outcome.

## 2 Methods

### 2.1 Setting

This study is situated within the Italian context of welfare transformation, where increasing attention has been given to community-based and relational approaches to addressing social vulnerability. Central to this shift is the concept of relational proximity (34), understood as an approach that emphasizes active engagement, shared responsibility and the co-production of support between service providers and users. Such an approach is particularly relevant for young people in NEET situations, who often experience distance from formal institutions and limited trust in traditional welfare and employment services.

Within this context, the C.O.P.E. (Capabilities, Opportunities, Places and Engagement) intervention was selected as a case study to explore how social prescribing can be experienced by young people in NEET conditions. The intervention combines principles of social prescribing with a community welfare perspective, aiming to address social, relational and wellbeing-related needs alongside pathways towards education or employment. Rather than focusing solely on rapid labor market activation, C.O.P.E. adopts a person-centered and flexible approach, tailored to participants’ individual circumstances and readiness. The C.O.P.E. intervention was piloted in Italy and Portugal between 2022 and 2024. In Italy, it was implemented within the Autonomous Province of Trento (APT), involving collaboration between public services and third-sector organizations. Currently the intervention has become a public service offered by APT. The broader project aimed to reach approximately 300 young people aged 16–34 in NEET conditions across the two countries, offering an opportunity to examine the intervention within different welfare and institutional settings. This study focuses specifically on the Italian pilot and on participants’ lived experiences of the intervention.

### 2.2 Data collection

A qualitative study design was adopted to explore participants’ experiences of the C.O.P.E. intervention from their own perspectives. Fifteen service users participating in the Italian pilot were recruited using maximum variation sampling, with the aim of capturing a diverse range of experiences across age, gender, geographical location and educational background. This sampling strategy was chosen to reflect the heterogeneity of the NEET population and to avoid over-representation of specific profiles. The sample size was guided by principles of information power and thematic saturation. Data collection continued until no substantially new themes emerged from the interviews, and the final interviews confirmed and consolidated existing patterns. This approach is consistent with qualitative research standards, where sample size is determined by the depth and richness of the data rather than numerical representativeness.

Data were collected through semi-structured interviews conducted between September 2023 and January 2024. Interviews took place either in person or online, according to participants’ preferences, in order to reduce barriers to participation. An interview guide was used to explore participants’ pathways into the project, their experiences of support, their relationship with the Link Worker, and perceived changes related to wellbeing, social engagement and work-related aspirations.

Ethical approval for the study was obtained from the C.O.P.E. scientific committee. Participants were contacted through their Link Workers, who informed them about the study and invited them to take part. Participation was entirely voluntary, and individuals who expressed interest were subsequently contacted by the research team to arrange the interview. No financial or material incentives were provided for participation. All participants received information about the study and provided informed consent prior to participation. To minimize potential power imbalances, interviews were conducted by researchers who were not directly involved in delivering the intervention.

### 2.3 Transcription, data management and analysis

All interviews were digitally audio-recorded with participants’ consent and lasted between 30 and 60 min. Recordings were transcribed verbatim and anonymized prior to analysis. Data were managed using the open-source qualitative analysis software Taguette (35).

Thematic analysis was conducted following an inductive approach, allowing themes to emerge from participants’ accounts rather than being predefined (36). An initial coding framework was developed based on the interview guide and iteratively refined as analysis progressed. Line-by-line coding was applied across the entire dataset to ensure systematic engagement with the data. Emerging themes and coding decisions were discussed within the research team to enhance analytical rigor and reflexivity. Coding was conducted by one researcher. To enhance analytical rigor, coding decisions and emerging themes were regularly discussed within the research team. These discussions supported the refinement of the coding framework and helped to ensure consistency and credibility in the analytical process. Themes were generated through an iterative process of grouping codes into broader patterns of meaning and comparing these across interviews. Themes were reviewed and refined through repeated engagement with the data to ensure internal coherence and clear distinctions between themes. Where uncertainties arose, the data were revisited and interpretations were discussed within the research team. To enhance analytical rigour, regular team meetings were held to reflect on emerging interpretations, challenge assumptions and support the development of robust and well-grounded themes. The research team had backgrounds in health management and public health. The researcher conducting the analysis was not involved in the delivery of the intervention. Reflexive discussions were undertaken throughout the analysis to consider potential assumptions and influences on interpretation.

The analysis focused on identifying recurring patterns in participants’ narratives concerning their engagement with the intervention, perceived benefits and challenges, the role of relational support, and the ways in which the intervention shaped their sense of agency and future orientation. Findings are presented thematically in the Results section, structured around key dimensions of participants’ experiences. The main themes identified are summarized in Table 1.

**Table 1:** Overview of themes identified in the analysis.

Theme	Description	Key dimensions
Entering the project	Initial engagement characterized by low expectations and external motivation	Family/professional referral; tentative participation; low intrinsic motivation
Relationship with labor market	Labor market experienced as uncertain and emotionally challenging	Fear of failure; low self-confidence; disorientation; perceived instability
The Link Worker support	Central relational figure bridging emotional support and employment guidance	Trust-building; flexibility; non-judgmental support; “in-between” role
Support received	Combination of practical guidance and emotional protection	CV/job search support; personalized pathways; pace aligned with wellbeing
Perceived value	Outcomes understood as gradual and psychosocial rather than immediate employment	Increased self-esteem; agency; clarity; future orientation
Who might benefit	Intervention as a preparatory and relational space for re-engagement	Psychosocial readiness; non-linear pathways; interaction of structural and individual factors

## 3 Results

### 3.1 Participant characteristics and profiles

Fifteen young people participating in the Italian C.O.P.E. pilot who met the NEET definition took part in the study. Participants ranged in age from 16 to 34 years and included ten men and five women. As shown in Table 2, they represented a heterogeneous group in terms of duration of NEET status, educational background and engagement with formal services. While some participants were already supported by social, mental health or employment services, others had no prior contact with institutional support and entered the project through self-referral or family encouragement. This diversity reflects the fragmented and non-linear trajectories characteristic of young people in NEET situations. The sample included more men than women (10 and 5 respectively). While the number of female participants was smaller, their experiences reflected key profiles commonly identified among young women in NEET situations, including trajectories shaped by social isolation, care responsibilities and mental health challenges. No substantial gender-specific patterns emerged in relation to the core themes identified; however, given the limited sample size, potential gender differences should be interpreted with caution.

**Table 2:** Participants profiles and characteristics.

NEET	Gender	Age	in NEET condition	Level of education	Services involved	N. sessions	Referral
NEET 1	Male	26	1 year	Secondary school	Community Mental Health Centre, Social workers, Substance Abuse	12	Rehabilitation technician at Community Mental Health Centre
NEET 2	Male	24	Few months	Middle-school	None	4	Parents, Public Employment Service
NEET 3	Male	20	Few months	Middle-school	None	14	Public Employment Service
NEET 4	Female	29	4 years	Secondary school	Social Work	6	Social Worker
NEET 5	Female	27	10 years	Secondary school	None	14	Parents
NEET 6	Male	34	1 year	Secondary school	None	4	Relative, self-referral
NEET 7	Male	18	2 years	Middle school	None	8	Parents
NEET 8	Male	34	10 years	Middle-school	Community Mental Health Centre, Social workers	4	Psychiatrist, Social Workers
NEET 9	Female	16	Few months	Middle-school	None	6	Public Employment Service
NEET 10	Female	21	3 years	Secondary school	Social Worker	6	Social Worker, Mother
NEET 11	Female	21	3 years	Middle-school	Residential psychiatric facilities	7	Educator
NEET 12	Male	22	3 years	Middle-school	None	10	Social Worker
NEET 13	Male	16	1 year	Middle-school	Criminal Justice System	3	Educator
NEET 14	Male	25	4 years	Secondary school	Community Mental Health Centre	6	Psychiatrist
NEET 15	Male	29	10 years	Middle-school	Social Worker	14	Social Worker

Adapted from Bertotti et al. (33).

### 3.2 Entering the project: low expectations and external motivation

Participants described highly diverse pathways into the C.O.P.E. intervention. A recurring theme concerned low initial expectations and limited intrinsic motivation at the point of entry that was mainly pressured by family members or professionals. Despite this initial hesitation, participants frequently described a sense of openness to “trying something different,” especially when feeling overwhelmed by prolonged inactivity or social isolation.

*“At first, I said OK, I’ll go to the first meeting, I’m doing it more for my parents than for myself.*

(NEET 5, female aged 27)

This suggests that engagement with the intervention often began as a tentative or exploratory step rather than a deliberate or goal-oriented choice. For some, participation represented an opportunity to interrupt a period of stagnation, even in the absence of clear goals or anticipated outcomes.

*I said it’s enough for me to do something I said, let us see, let us try, but I honestly did not expect it.*

(NEET 4, aged 29)

Overall, these accounts indicate that initial engagement with the intervention was shaped by a combination of external prompting and a situational readiness to re-engage, rather than by strong intrinsic motivation. This highlights the importance of low-threshold and relational entry points for reaching young people who may be distant from formal support systems.

### 3.3 Relationship with labor market: fear, disorientation and low confidence

Across interviews, participants described a strained and often distressing relationship with the labor market prior to the intervention. Work was frequently portrayed not as a source of opportunity, but as a threatening and pressurizing environment, particularly for those with limited work experience or fragile mental health. This suggests that labor market disengagement was not only a structural condition, but also a deeply internalized experience shaping participants’ sense of self. Low self-esteem emerged as a central barrier, especially among participants who had never worked or who had experienced repeated failures. Participants often framed their lack of confidence as both a consequence and a reinforcement of their disengagement.

*“Maybe you just try once, or twice. Maybe some places tell you no, you did not pass the test. If you do not even give me time to learn, see how I work.*

(NEET 6, aged 34)

Participants also expressed a broader sense of confusion and lack of guidance in navigating the transition to adulthood. Work was perceived as unstable, unpredictable and difficult to access, contributing to feelings of paralysis and uncertainty about the future. In this sense, disengagement appeared to be shaped not only by personal vulnerabilities, but also by wider perceptions of structural insecurity.

*“We grew up in the opposite world without any security, you go into crisis. So many young people are just like me who do not study, do not work because you do not even know where to start.*

(NEET 5, aged 27)

### 3.4 The link worker support: an “in between” and relational role

Participants consistently described the Link Worker as occupying an “in-between” role, situated between psychological support and employment counselling. This hybrid positioning was perceived as a key strength of the intervention, differentiating it from other services participants had previously encountered. In this sense, the Link Worker appeared to bridge a gap between psychosocial support and practical orientation, responding to needs that were often unmet within more fragmented service systems.

“*Having a person there specifically for you who is somewhere between a psychologist and an employment center.*

(NEET 5, aged 27)

The relational dimension of the support was particularly valued. Participants emphasized the informal, non-judgmental and listening approach adopted by Link Workers, often contrasting it with the more goal-oriented and time-pressured nature of other services. Compared to other professionals, the Link Worker was perceived as having greater flexibility and fewer institutional constraints, allowing for a more personalized and responsive form of support.

*“The social worker and the LW are not so different, but there are differences. The social worker listens less to the young person. Whereas LWs have much more flexibility and are given this space where they can help the young person, just be of help to the young person without certain goals.*

(NEET 14, aged 25)

Overall, these findings highlight how the effectiveness of the intervention was closely linked to its relational and flexible nature. The Link Worker functioned as a mediating figure capable of aligning emotional support with practical guidance thereby facilitating a form of engagement that was both accessible and responsive to participants’ readiness and needs.

### 3.5 Support received: combining practical guidance and emotional protection

While the relational aspect created the conditions for engagement, participants also highlighted the practical value of the support received. The intervention provided step-by-step guidance in navigating work-related processes, such as writing a CV, preparing for interviews and identifying suitable job opportunities. This suggests that practical support was most effective when embedded within a trusting and supportive relationship.

*“The project stays behind the young people, they help them with this somewhat targeted search, let us say, a bit more focused, a bit more attentive to their own needs as well.*

(NEET 5, aged 27)

Importantly, this practical support was delivered with careful attention to participants’ emotional and psychological conditions. Several participants emphasized the importance of progressing at a pace that would not undermine their wellbeing, particularly in the context of anxiety, low confidence or previous negative experiences with work. In this sense, employment-related support was not experienced as a pressure to achieve immediate outcomes, but as a gradual and protected process.

*“Not only finding what you like but above all finding a place that does not make you go back on your steps, so do not destroy what you have already created because otherwise it would be detrimental to the person.*

(NEET 14, aged 25)

For those who have no support network the Link Worker also represented a key point of reference in decision-making processes. The availability of a consistent and trusted figure contributed to reducing uncertainty and supported participants in navigating both personal and employment-related choices.

Overall, these accounts highlight how the intervention combined practical guidance with emotional protection, enabling participants to engage with employment-related activities in a way that was aligned with their readiness and psychological stability.

### 3.6 Perceived value of the intervention: agency, direction and renewed engagement

Participants consistently described the outcomes of the intervention in terms that extended beyond immediate employment. Rather than leading directly to job placement, the intervention was experienced as supporting a process of gradual personal and social re-engagement. This suggests that its primary effects were psychosocial, creating the conditions necessary for future engagement with education or work. Participants emphasized improvements in self-esteem, confidence and sense of direction. For many, the intervention contributed to a shift from feelings of stagnation and disorientation towards a greater sense of agency and possibility.

*“From 2014 to this year, for the last 10 years, I was in limbo, for me to start with this project was almost a rebirth.*

(NEET 5, aged 27)

These changes were often described as subtle but meaningful, particularly in the context of prolonged inactivity or previous negative experiences. Rather than representing discrete outcomes, they reflected ongoing processes of rebuilding confidence, clarifying personal goals and developing a more positive orientation towards the future.

Importantly, participants did not frame employment as an immediate or singular objective. Instead, work was understood as one component of a broader process of stabilization and personal development. In this sense, the intervention appeared to support what can be understood as psychosocial readiness for labor market engagement, enabling participants to approach employment-related opportunities with greater confidence and awareness.

For some participants, this translated into concrete steps, such as re-engaging with training, exploring job opportunities or considering new pathways. However, these developments were generally described as gradual and contingent on individual readiness, rather than as direct outcomes of the intervention.

Overall, these findings highlight how the value of the intervention lies not primarily in immediate employment outcomes, but in its capacity to support processes of psychosocial stabilization, agency development and progressive re-engagement with social and employment pathways.

*“Certainly, the link worker helped more than the agency of work. [...] It helped me to understand even myself.*

(NEET 6, aged 36)

### 3.7 Who might benefit from the intervention: early support and intervention

Taken together, the findings highlight how participants’ engagement with the intervention was shaped by an interplay between psychological vulnerability, relational support and structural conditions. Rather than following a linear pathway towards employment, participants’ trajectories were characterized by gradual and often non-linear processes of re-engagement.

Across themes, a consistent pattern emerged in which initial low motivation and uncertainty were mediated by relational forms of support, particularly through the role of the Link Worker. This relational dimension appeared to enable participants to stabilize their emotional conditions, rebuild confidence and progressively engage with practical steps related to education or employment.

At the same time, participants’ accounts point to the limits of approaches that prioritize rapid labor market activation. Experiences of fear, low self-confidence and perceived labor market insecurity suggest that immediate employment may not be a realistic or appropriate objective for many young people in NEET situations. Instead, engagement appears to depend on the development of intermediate processes, including trust, agency and psychosocial stability.

*“It could be a very useful support because even in schools we need to raise awareness not only of mental health but health in general.*

(NEET 1, aged 26)

In this sense, the intervention can be understood as operating within a preparatory space, where relational and psychosocial dimensions are prioritized as preconditions for future engagement. This highlights the importance of aligning support with participants’ readiness, rather than imposing standardized or outcome-driven pathways.

Overall, the findings suggest that social prescribing interventions targeting young people in NEET situations may be most effective when they combine relational support with gradual and personalized pathways, acknowledging the complex interaction between individual experiences and broader structural constraints.

*“I would still be standing. My anxiety and panic disorder even worsen. So it would probably always get worse or even go to a psychiatric center for sure. But thanks to this project I’ve escaped it for now.*

(NEET 5, aged 27)

## 4 Discussion

This study provides one of the first qualitative accounts of young people’s perspectives on participating in a social prescribing intervention in Italy that explicitly addresses pathways towards labor market engagement. The results highlight the heterogeneity of NEET trajectories and confirm that disengagement from education and employment cannot be understood solely in terms of labor market barriers. Participants’ accounts illustrate how social isolation, low self-esteem, mental health difficulties and previous negative experiences with work intersect, shaping both their distance from the labor market and their readiness to re-engage. In this sense, the study reinforces the idea that health and employment are deeply interconnected, rather than separate domains, and that interventions addressing only one dimension risk being insufficient for the most vulnerable young people.

These findings provide empirical support for the conceptual framework outlined in the introduction, illustrating how structural, relational and individual dimensions interact in shaping NEET trajectories. In particular, the results highlight the central role of relational mechanisms in mediating the effects of structural constraints and supporting the development of individual agency.

Two broad profiles emerged among participants: young people already engaged with social or mental health services, and those experiencing high levels of isolation without formal support. While these groups entered the intervention through different pathways, both described fragile relationships with the labor market characterized by fear, confusion and low confidence. This finding suggests that social prescribing may function as a flexible and inclusive entry point, capable of reaching young people who are either over-served by fragmented systems or entirely disconnected from them.

A central contribution of this study concerns the role of the Link Worker. Consistent with early evidence from social prescribing initiatives targeting NEET populations, the Link Worker emerged as a key relational figure who supports young people through a stepped and personalized approach. Rather than acting as a direct mechanism for labor market activation, the Link Worker appeared to facilitate a preparatory process, combining emotional containment with practical guidance and supporting the gradual rebuilding of agency, confidence and future orientation. At the same time, these findings point to the importance of considering how relational dynamics interact with broader institutional structures. While the Link Worker plays a central role in facilitating engagement, their effectiveness is shaped by the degree of coordination between health, social and employment services. In fragmented systems, relational support may compensate for institutional gaps, whereas more integrated service configurations may enhance its impact. This suggests that social prescribing should be understood not only as a relational intervention, but as part of wider service ecosystems (37).

This finding aligns with recent evaluations of social prescribing interventions for NEETs, which emphasize relational mechanisms and contextual factors over linear employment outcomes (38). Participants’ narratives also illuminate how the value of the intervention lies in its capacity to create a protected and non-judgmental space in which wellbeing and employment-related concerns can be addressed simultaneously. In contrast to traditional employment services, which were often perceived as pressurizing or misaligned with their emotional readiness, C.O.P.E. was experienced as allowing time, flexibility and reflection. This challenges narrow interpretations of labor market activation and supports a broader understanding of social prescribing as an enabling space that precedes, rather than replaces, engagement with work or education. Similar dynamics have been observed in recent studies on social prescribing with young people, which highlight the importance of trust, continuity and relational support in fostering engagement and wellbeing (39).

Importantly, participants themselves emphasized that the benefits of the intervention were not limited to concrete employment outcomes. Instead, they described increased clarity, motivation and a renewed sense of possibility, suggesting that social prescribing may contribute to preventing further disengagement and deterioration of mental wellbeing. This should be interpreted as reflecting participants’ perceived experiences and subjective changes, rather than measurable employment outcomes. While the intervention appears to support processes of re-engagement, the qualitative design does not allow causal inference regarding labor market participation. This finding is particularly relevant in light of the health–work paradox, as it illustrates how supporting mental wellbeing and social connection may represent a necessary precondition for sustainable labor market participation. From a policy perspective, these findings suggest the need to integrate relational and psychosocial forms of support within employment services, particularly through early-stage and low-threshold interventions targeting young people with complex needs.

From a policy perspective, these findings suggest the need to integrate relational and psychosocial forms of support within employment services, particularly through early-stage and low-threshold interventions targeting young people with complex needs.

## 5 Conclusion

This study explored young people’s perceptions of a social prescribing intervention targeting NEETs within the Italian context. By foregrounding users’ voices, it contributes to existing literature by illustrating how social prescribing can operate as a relational and preparatory space, bridging the divide between wellbeing support and pathways towards education or employment.

The findings suggest that, for young people in NEET situations, readiness for labor market engagement is closely intertwined with psychosocial wellbeing, confidence and social connection. Rather than offering a direct or instrumental route into employment, the C.O.P.E. intervention appears to support gradual re-engagement by fostering agency, trust and self-understanding. In doing so, it challenges rigid distinctions between health-focused and employment-focused interventions and highlights the potential of more integrated, youth-sensitive welfare approaches.

From participants’ perspectives, the value of the intervention also lies in its preventative potential, particularly if offered earlier in life during key transition phases. These insights point to the importance of designing social prescribing initiatives that recognize heterogeneity among NEETs and prioritize relational continuity and flexibility over standardized activation measures.

### 5.1 Limitations and future research

This study has several limitations that should be considered when interpreting the findings. First, the sample size was relatively small (*n* = 15), which is consistent with qualitative research but limits the range of perspectives captured. Although maximum variation sampling was used, the findings cannot be considered representative of the broader NEET population.

Second, the study focuses on a single geographic context, the Autonomous Province of Trento (Italy), and findings may therefore not be directly transferable to other settings with different institutional and labor market conditions.

Third, the study is subject to potential selection bias, as participants were recruited among individuals who were already engaged in the intervention and willing to take part in the interviews. This may have led to an over-representation of more positive or engaged experiences, while the perspectives of those who disengaged early or chose not to participate are not captured.

Fourth, the study is based on cross-sectional qualitative data and does not include longitudinal follow-up. As a result, it does not allow assessment of longer-term outcomes, particularly in relation to sustained engagement with education or employment. The findings should therefore be interpreted as reflecting participants’ perceptions and experiences rather than measurable long-term impacts.

Finally, as in all qualitative research, the analysis is subject to researcher interpretation. Although steps were taken to enhance analytical rigor, including iterative coding and team discussions, the findings may have been influenced by the researchers’ perspectives.

Future research could build on these findings by adopting longitudinal and mixed-methods approaches, and by including the perspectives of additional stakeholders, such as Link Workers and service providers, to better understand how social prescribing operates across different contexts.

## Data Availability

The raw data supporting the conclusions of this article will be made available by the authors, without undue reservation.
